# Autophagy is required for human umbilical cord mesenchymal stem cells to improve spatial working memory in APP/PS1 transgenic mouse model

**DOI:** 10.1186/s13287-017-0756-2

**Published:** 2018-01-15

**Authors:** Wen Li, Kai Li, Jing Gao, Zhuo Yang

**Affiliations:** 1 School of Medicine, State Key Laboratory of Medicinal Chemical Biology, Key Laboratory of Bioactive for Materials Ministry of Education, Nankai University, 94 Weijin Road, Tianjin, 300071 China; 2 Tianjin Third Central Hospital, Tianjin, 300170 China

**Keywords:** Autophagy, Human umbilical cord mesenchymal stem cells, Long-term potentiation, Neurogenesis, Synaptic formation

## Abstract

**Background:**

Recent studies have shown that autophagy plays a central role in mesenchymal stem cells (MSCs), and many studies have shown that human umbilical cord MSCs (huMSCs) can treat Alzheimer’s disease (AD) through a variety of mechanisms. However, no studies have looked at the effects of autophagy on neuroprotective function of huMSCs in the AD mouse model. Thus, in this study we investigated whether inhibition of autophagy could weaken or block the function of huMSCs through in vitro and in vivo experiments.

**Methods:**

In vitro we examined huMSC migration and neuronal differentiation by inhibiting or activating autophagy; in vivo autophagy of huMSCs was inhibited by knocking down Beclin 1, and these huMSCs were transplanted into the APP/PS1 transgenic mouse. A series of related indicators were detected by T-maze task, electrophysiological experiments, immunofluorescence staining, enzyme-linked immunosorbent assay (ELISA), and Western blotting.

**Results:**

We demonstrated that regulation of autophagy can affect huMSC migration and their neuronal differentiation. Moreover, inhibition of autophagy in huMSCs could not realize neuroprotective effects via anti-apoptosis or promoting neurogenesis and synapse formation compared with those of control huMSCs.

**Conclusions:**

These findings indicate that autophagy is required for huMSCs to maintain their function and improve cognition impairment in APP/PS1 transgenic mice.

## Background

Autophagy plays a key role in normal physiology and pathology; it degrades cell organelles and misfolded proteins by fusing autophagosomes with lysosomes to prevent waste accumulation and achieves intracellular homeostasis and cell organelle self-renewal. Autophagy is administered by a series of well-characterized proteins: for example, Beclin 1 is responsible for the initiation of the autophagosome; ATG and LC3 members are responsible for the elongation and formation of the autophagosome; and P62 is a polyubiquitin-binding protein that is incorporated into the autophagosome and undergoes degradation in autolysosomes, and is thus inversely related to the autophagy level [1]. Abnormal expression of these proteins will lead to failure of the autophagy process and some diseases such as cancer, neurodegenerative disease, and immune disease.

Human umbilical cord mesenchymal stem cells (huMSCs) are stromal cells isolated from the fetal umbilical cord with Wharton’s jelly. huMSCs are positive for CD29, CD44, and CD90, and negative for CD31, CD34, and CD45 [2], and in line with the characteristics of stem cells. Mesenchymal stem cells (MSCs) are multipotent and can be differentiated into various cells of mesodermal lineage in vitro [3–5]. In vivo, MSCs have the ability to migrate to sites of injury and differentiate as well as releasing trophic and growth factors to protect tissue from damage [6–8]. Recent studies have shown that autophagy could regulate the differentiation of MSC-derived cell lineages, stemness maintenance, and cell senescence [9–11]. For instance, autophagy promoted the differentiation of MSCs into neurons [12] and osteoblasts [13]. Rapamycin-induced autophagy contributed to maintaining MSC stemness, while using 3-methyladenine (3-MA) to inhibit autophagy led to loss of stemness. Similarly, inhibition of autophagy with 3-MA leads to a reduced expression of senescence-related proteins in the process of MSC senescence [14, 15]. Moreover, there is a close relationship between autophagy and apoptosis [16, 17]. The autophagic process serves a physiologic function to maintain cellular viability and delays cell apoptosis during periods of starvation. A previous study has shown that autophagy prevents MSC apoptosis under hypoxia/serum deprivation, and inhibition of autophagy allows MSCs to exhibit higher rate of apoptosis [18]. In summary, autophagy is crucial for the fate and function of MSCs.

Alzheimer’s disease (AD) is the most common neurodegenerative disorder. It is caused by: 1) synapse loss, such as PSD95 expression decrease; 2) neuronal apoptosis, which leads to abnormal brain function; and 3) the presence of amyloid-β (Aβ) plaques and tau tangles, which result in learning and memory impairment [19, 20]. Aβ is regulated by amyloid precursor protein (APP), and failure to clear Aβ will lead to a series of downstream events in AD. Recent research has proposed that MSC transplantation provides neuroprotective effects for neurodegenerative disorders [21, 22]. Furthermore, in vivo MSCs can affect the recovery of cognitive function through complex mechanisms such as secreted neurotrophic factors [23], increased neurogenesis [24], and reduced neuronal apoptosis. However, no studies have investigated what effect these functions of MSCs have in AD. In this study, we investigated whether inhibition of autophagy could weaken or arrest the function of huMSCs, and whether inhibition of autophagy in huMSCs transplanted into an AD mouse model can exert neuroprotective effects or generate worse effects through in vitro and in vivo experiments.

## Methods

### huMSC cultures, drug treatments, and lentiviral transfection

Human umbilical cords were obtained from full-term births after either cesarean section or normal vaginal delivery with the consent of parents in Tianjin First Center Hospital, Tianjin, China. The procedure of primary huMSC separation was according to Yang et al. [19]. huMSCs were cultured in Dulbecco’s modified Eagle’s medium (DMEM-F12; HyClone) containing 10% fetal bovine serum (FBS; Sigma-Aldrich, F2442). huMSCs underwent three passages for this study. huMSCs were co-treated with 20 mg/ml tricyclodecane-9-yl-xanthogenate (D609; Sigma-Aldrich, T8543) [25] and 10 ng/ml rapamycin [26] (Rap; Sigma-Aldrich, 37094) or 5 mmol 3-MA [27] (Sigma-Aldrich, M9281) in a humidified incubator at 37 °C and 5% CO_2_ for 2 h and 4 h. huMSCs were then collected for assay. All experiments were replicated three times.

To investigate the migration ability of huMSCs, the cells were cultured in six-well plates and a wound was created by scratching with a sterile plastic pipette tip. huMSCs were then treated with 10 ng/ml Rap or 5 mmol 3MA in DMEM-F12 without FBS for 24 h. After incubation, the cells were washed with phosphate-buffered saline (PBS) and the migrated cells of the wound area were observed by microscope (Olympus). The area of cell migration was counted by ImageJ software.

Beclin-1 levels of huMSCs were knocked down by a lentivirus containing small-hairpin (sh)RNA and green fluorescent protein (GFP) reporter gene. The lentivirus was purchased from GeneChem (Shanghai, China). The following nucleotide sequences were used for the cloning of shRNA encoding sequences into a lentiviral vector: Beclin-1 (Becn 1): 5′-ccggga CAGTTTGGCACAATCAATACTCGAGTATTGATTGTGCCAAACTGTCTTTTTg-3′; and negative controls (NC): 5′-CCGGTTCTCCGAACGTGTCACGTTTCAAGAGAACGTGACACGTTC GGAGAATTTTTG-3′. huMSCs were stably infected with negative control lentivirus (huMSCs-shNC) or lentivirus expressing shRNA inhibiting the gene Beclin-1 (huMSCs-shBecn 1).

#### Animals

Heterozygous APPswe/PS1dE9 double-transgenic male mice (6 months old) were bred with background-matched C57BL/6 mice; this type of transgenic mouse has been widely used [28, 29] and exhibits early Aβ accumulation which is a typical characteristic in AD. All mice were purchased from Beijing HFK Bio-Technology Co. Ltd. (Beijing, China).

### huMSC transplantation in APP/PS1 double-transgenic mice

huMSCs-shNC and huMSCs-shBecn 1 were suspended in saline at a density of 2 × 10^5^ cells/μl. Mice were anesthetized with an intraperitoneal injection of chloral hydrate (0.4 g/kg; Sigma-Aldrich), and 5 μl of saline or huMSCs-shNC or huMSCs-shBecn 1 suspension was then injected into the left lateral ventricles (0.1 mm caudal, 0.9 mm bilateral to bregma, and 2.0 mm ventral from the dura mater) of the brain at a delivery rate of 1 μl/min using a 10-μl Hamilton microsyringe fixed on a stereotaxic apparatus (Narishige, Japan). After the injection, the needle was kept in place for 5 min before it was slowly retracted. The animals were divided into three groups (*n* = 6): 1) the AD-Veh group—APP/PS1 mice were subjected to saline; 2) huMSCs-shNC group—APP/PS1 mice were subjected to huMSCs-shNC suspension; and 3) huMSCs-shBecn 1 group—APP/PS1 mice were subjected to huMSCs-shBecn 1 suspension. All mice were sacrificed on post-transplantation day 14.

### Spatial working memory on the elevated T-maze

To assay working memory performance, a T-maze task was employed. The protocol followed that of Deacon and Rawlins [30]. The T-maze consisted of a start arm (30 × 10 cm) and two goal arms (30 × 10 cm), surrounded by a 20-cm high wall. In brief, prior to the start of the formal experiment, the body weight of the mice was reduced to 90% of their original weight by restricting food intake. Then followed the habituation phase, when mice became adapted to the T-maze, and condensed milk as a reward (0.07 ml/reward; Nestle) was given in the food well at the end of the arm. During the trial phase, each trial consisted of a forced choice and a free choice. For the forced choice, one of two goal arms was blocked by a wall and the mouse was directed towards the open arm with a condensed milk reward, and then the mouse was returned to the start box. For the choice phase, the wall was removed and the mouse had to select the formerly closed arm to receive a second reward [31] (this was the rewarded alternation and recorded as correct, if not it was recorded as wrong) (Fig. 3a). In this study, the time interval between the forced choice and the free choice was approximately 1 min. Mice were subjected to 10 trials per day for 4 consecutive days. A percentage of correct choices per animal was calculated.

### Long-term potentiation (LTP) and depotentiation (DEP) recordings

Following the T-maze test, LTP and DEP were assessed by an in vivo electrophysiological test, based on previous studies [32]. The mouse was anesthetized with an intraperitoneal injection of urethane (1.2 mg/kg; Sigma-Aldrich) and then positioned on the stereotaxic apparatus prepared for surgery. First, the skull was exposed and a hole was drilled for inserting electrodes. Then the bipolar stimulating electrode was positioned in the performant pathway (PP; 2.1 mm lateral and 3.8 mm posterior to the bregma, 1.8 mm from the brain surface) and the monopolar stainless steel recording electrode was positioned in the dentate gyrus (DG; 1 mm lateral and 1.7 mm posterior to the bregma, 1.8 mm from the brain surface) of the hippocampus. The stimulation intensity (range 0.3–0.5 mA, stimulus pulse 0.2 ms at 0.03 Hz) was used to stimulate a response at 70% of its maximum to deliver baseline, LTP, and DEP recordings (Scope software, PowerLab; AD Instruments, New South Wales, Australia). The baseline was recorded every 30 s for 20 min. After the baseline, theta burst stimulation (TBS) was delivered to induce LTP, and then the same single plus stimulating intensity was recorded every 60 s for 1 h as LTP. Following LTP, low-frequency stimulation (LFS; 900 pulses, 1 Hz for 15 min) was delivered to induce DEP, and the same method as used for recording LTP was used to record DEP. The field excitatory postsynaptic potential (fEPSP) slope was measured by Clampfit 10.0 (Molecular Devices, Sunnyvale, CA, USA).

### Histology examination

Mouse brain tissue was embedded in OCT compound (Tissue-Tek, Miles) and sectioned at 10-μm intervals (Leica CM 1850). The sections were stained with hematoxylin and eosin (H&E). Survival of neurons in the hippocampus and cortex was calculated from six sections of each sample, and the average was taken. Microscopic images were analyzed by ImageJ software.

### Immunofluorescence staining

Brain sections and cultured huMSCs were fixed in 4% paraformaldehyde for 10 min and then incubated with 0.5% Triton-X100 for 10 min. After each step, the sections were washed three times with PBS. The sections were blocked with 10% normal goat serum for 1 h at room temperature, followed by incubation with the primary antibodies rabbit anti-NSE (1:100, Abcam, ab53025), rabbit anti-MAP2 (1:500, Abcam, ab32454), mouse anti-Human Nuclear Antigen (hNu; 1:200, Abcam, ab191181), mouse anti-LC3 (1:1000, MBL, M186-3), rabbit anti-Cleaved Caspase-3 (CCaspase-3; 1:500, Cell Signaling, 9661), rabbit anti-Sox2 (1:1000, Abcam, ab97959), mouse anti-SQSTM1/P62 (P62; 1:1000, Abcam, ab56416), Rabbit anti-Aβ 1-42 (1:100, Bioss, China, bs-0107R), rabbit anti-DCX (1:500, Abcam, ab77450), rabbit anti-Ki67 (1:250, Abcam, ab16667), and rabbit anti-Postsynaptic density protein 95 (PSD95; 1:250, Abcam, ab16667) for 24 h at 4 °C. After washing three times with PBS, the sections were stained with the secondary antibodies Alexa 488-conjugated goat anti-mouse IgG (1:1000, CA11008S; Invitrogen) and Alexa 594-conjugated goat anti-rabbit IgG (1:1000, A21235; Life Technologies) for 1 h at room temperature. They were then washed three times with PBS. Subsequently, the nucleus was stained with DAPI (1:1000, Solarbio, China) for 5 min. Images were taken on a confocal laser scanning microscope (Olympus). The fluorescent value was quantified by ImageJ software, with six sections of each sample being calculated and the average taken.

### Western blot assay and enzyme-linked immunosorbent assay (ELISA)

Collected cell pellets and mouse brain lysates were prepared, and the procedure for Western blotting was performed as previously described [32]. The primary antibodies for the Western blot analysis were as follows: rabbit anti-Beclin 1 (1:1000, Cell Signaling, 3495), rabbit anti-ATG 7 (1:500, Cell Signaling, 2631), rabbit anti-SDF-1 (1:1000, Abcam, ab18919), rabbit anti-PARP (1:1000, Cell Signaling, 9542), rabbit anti-APP (1:5000, Abcam, ab180140), rabbit anti-Presenilin1 (PS1; 1:5000, Abcam, ab76083), rabbit anti-Bcl-xl (1:2000, Cell Signaling, 2764), rabbit anti-Bax (1:2000, Abcam, ab32503), rabbit anti-CaMKII (1:2000, Abcam, ab52476), rabbit anti-CaMKII (phospho) (p-CaMKII; 1:2000, Abcam, ab32503), rabbit anti-NMDAR2B (1:1000, Cell Signaling, 4212), rabbit anti-ACTB (β-actin; 1:5000, Sangon, China), and anti-LC3 antibody (1:1000), anti-MAP2 antibody (1:1000), anti-SQSTM1/P62 antibody (1:2000), anti-Sox2 antibody (1:1000), anti-CCaspase-3 antibody (1:1000), and anti-PSD95 antibody (1:2000) as previously described. The secondary antibodies were anti-rabbit IgG (H + L), HRP conjugate (1:5000, Promega) or anti-mouse IgG (H + L) HRP conjugate (1:5000, Promega). Immunoreactivity was obtained by a chemiluminescence imaging system (GE Healthcare, RPN2108), and ImageJ software was used to evaluate the differences between the samples.

Brain-derived neurotrophic factor (BDNF) and nerve growth factor (NGF) in the hippocampus and cortex levels were measured by an ELISA method using the mouse BDNF (SEA011Mu, Cloud-Clone Corp., China) or NGF (SEA105Mu, Cloud-Clone Corp., China) assay kits in accordance with the manufacturer’s instructions.

### Statistical analysis

For all animal experiments, rats were selected by a completely randomized design to each group. A double-blinding method was used for group assignment and outcome assessment. The method of assessing the sample size was according to our previous studies [33]. All results are expressed as mean ± standard error of the mean (SEM). Data were generated from three independent experiments. Statistical analysis was performed by SPSS 22.0 (SPSS Inc.) and GraphPad Prism 6 (GraphPad Software). One-way analysis of variance (ANOVA) followed by further Dunnet’s multiple comparison was used to analyze the statistical differences between three or more groups, and *P* < 0.05 was considered statistically significant.

## Results

### Autophagy promotes migration and neuronal differentiation of huMSCs in vitro

**Fig. 1:**
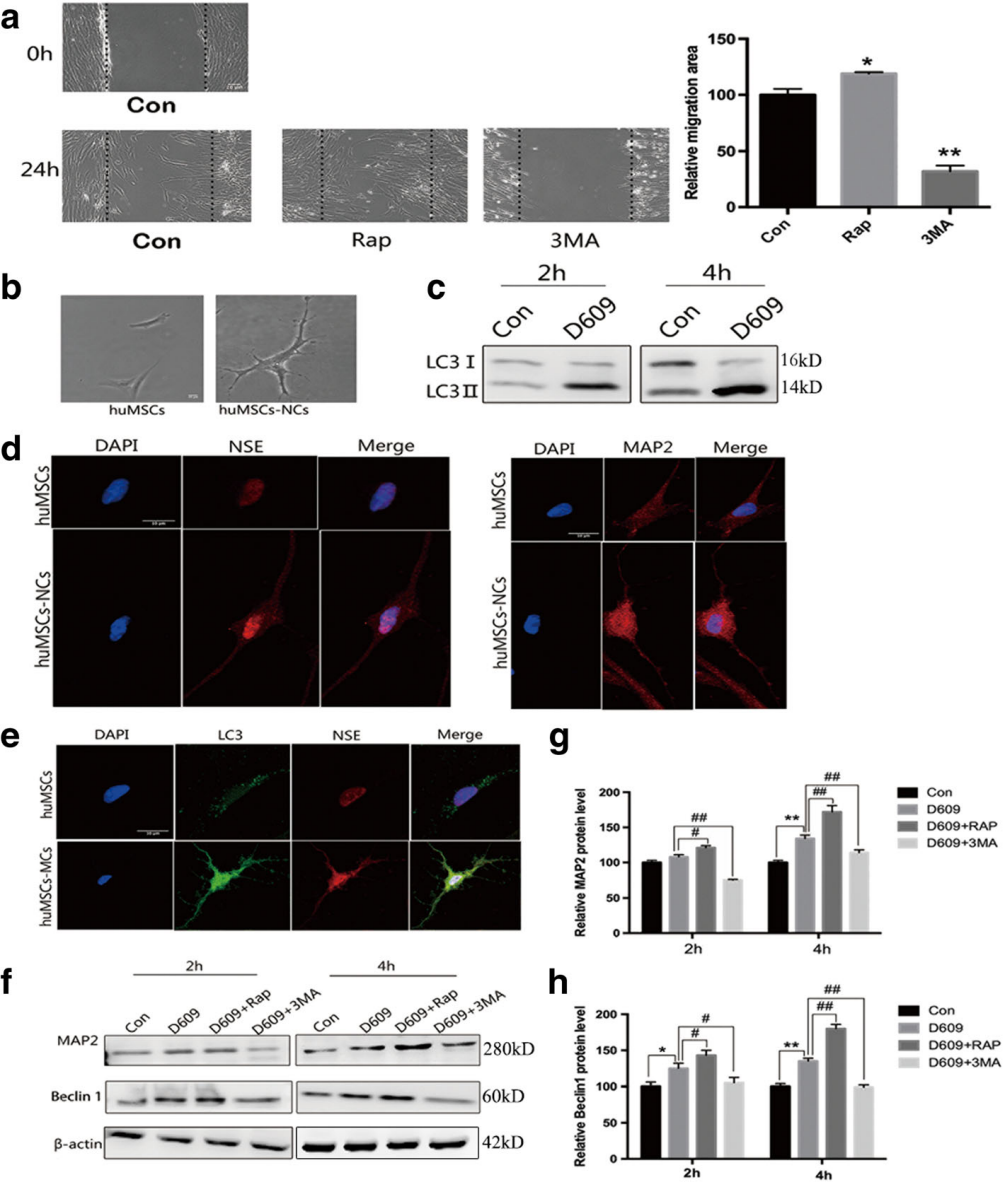
Autophagy promotes migration and neuronal differentiation of huMSCs in vitro. **a** The effect of autophagy on migration of human umbilical cord mesenchymal stem cells (huMSCs) with rapamycin (Rap) or 3-methyladenine (3MA) treatment (*n* = 3). **b** The morphology of huMSCs was changed to huMSCs-NCs with tricyclodecane-9-yl-xanthogenate (D609) treatment for 4 h. **c** Representative cropped Western blots of the autophagy marker LC3 II in huMSCs at 2 h and 4 h after treatment with D609. **d** The expression of the neuronal markers neuron-specific enolase (NSE) and microtubule-associated protein 2 (MAP2) in huMSCs and huMSCs-NCs. **e**–**h** The effect of autophagy on neuronal differentiation of huMSCs. **e** MAP2 and LC3 II were co-localized on double immunofluorescence staining. **f** Representative cropped Western blots of Beclin 1 and MAP2. Statistical analysis of **g** MAP2 and **h** Beclin 1 (*n* = 3). All data are expressed as mean ± SEM from three independent experiments. *P < 0.05, **P < 0.01, vs. control (Con); ^#^*P* < 0.05, ^##^*P* < 0.01, vs. D609

In order to investigate whether autophagy was involved in migration and neuronal differentiation of huMSCs, we first conducted a scratch test and treated huMSCs with Rap, an autophagy inducer, or 3MA, an autophagy inhibitor. The data showed that huMSC migration was significantly boosted by Rap and significantly inhibited by 3MA compared with the control group (Fig. 1a).

Previous studies have reported that D609 can induce MSC differentiation into neuron-like cells [25]. Therefore, in this study D609 was employed to treat huMSCs. It was found that the morphology of huMSCs became neuron-like (huMSCs-NCs) after treatment for 4 h (Fig. 1b); simultaneously, the expression of LC3 II (an autophagy marker) was significantly increased from 2 h to 4 h of treatment (Fig. 1c). To further understand the relationship between autophagy and neuronal differentiation of huMSCs, neuron-specific enolase (NSE) and microtubule-associated protein 2 (MAP2) (neuronal markers) and LC3 II were stained with their specific antibodies and observed using confocal microscopy. The increased expression of NSE and MAP2 indicated that huMSCs-NCs have the potential to function as neurons (Fig. 1d). Then MAP2 and LC3 II were co-localized by double immunofluorescence staining. The data showed that the expressions of MAP2 and LC3 in huMSCs-NCs were simultaneously increased (Fig. 1e). In addition, MAP2 and Beclin 1 (autophagy-related protein) were examined with Western blot assay. At 2 h and 4 h of huMSC differentiation, activation of autophagy with Rap can promote the expression of MAP2; in turn, inhibition of autophagy with 3MA prevented neuronal differentiation (Fig. 1f and g). Beclin 1 expression was consistent with MAP2 expression (Fig. 1f and h).

Taken together, these data imply that in vitro migration and neuronal differentiation of huMSCs is tightly regulated by the autophagy pathway.

### The effect of inhibition of autophagy on huMSC function in vitro

**Fig. 2:**
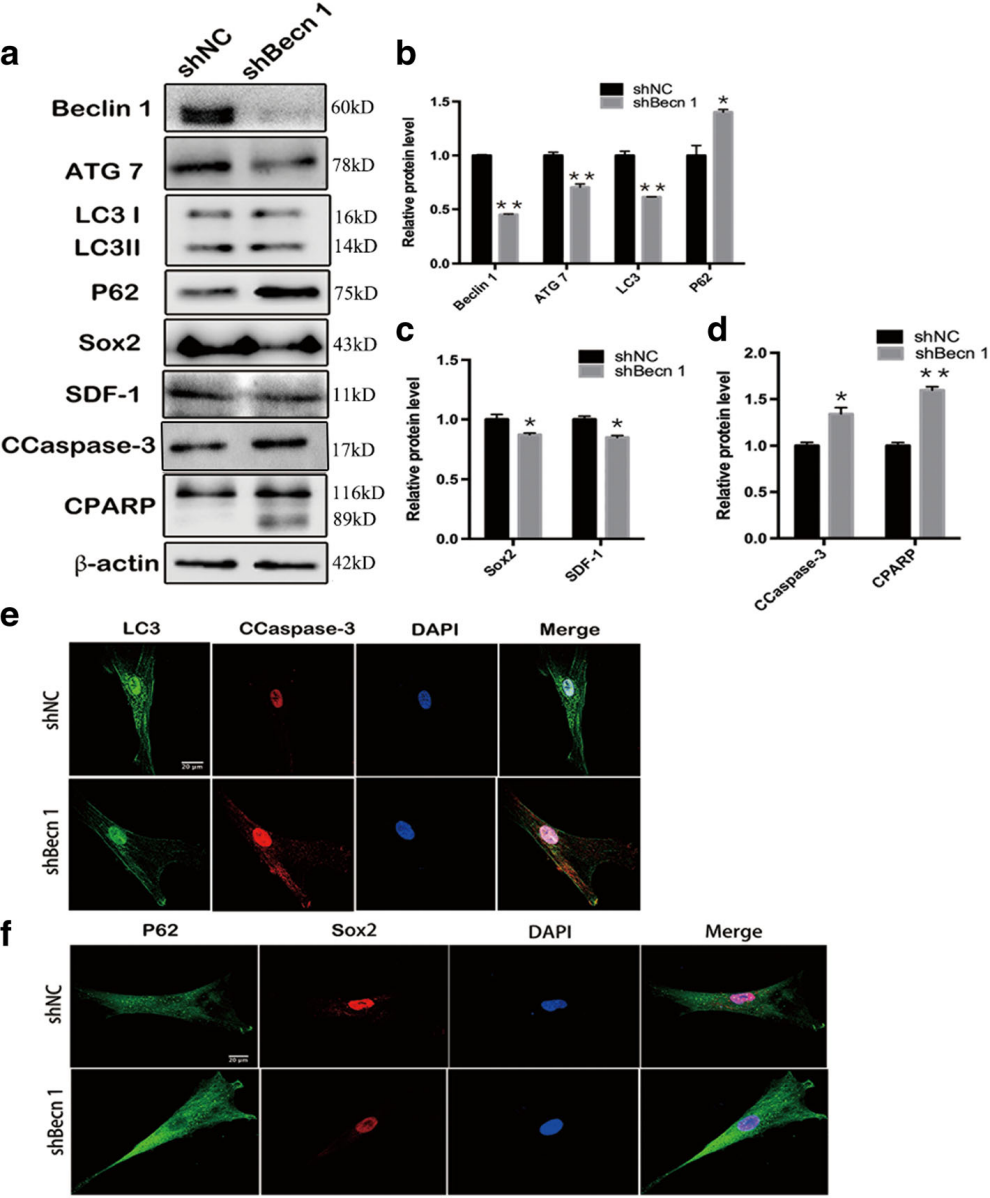
The inhibitory effect of autophagy on huMSC function in vitro. **a** Representative cropped Western blots of Beclin 1, ATG7, LC3, P62, Sox2, stem cell-derived factor-1 (SDF-1), cleaved caspase-3 (CCapase-3), and cleaved poly-ADP-ribose polymerase (CPARP). **b** Statistical analysis of autophagy-related proteins Beclin 1, ATG7, LC3, and P62 (*n* = 3). **c** Statistical analysis of migration- and differentiation-related proteins Sox2 and SDF-1 (*n* = 3). **d** Statistical analysis of apoptosis-related proteins Capase-3 and PARP (*n* = 3). **e**,**f** LC3 and CCaspase-3, P62, and Sox2 were co-localized by double immunofluorescence staining, respectively. All data are expressed as mean ± SEM from three independent experiments. *P < 0.05, **P < 0.01, vs. shNC

To explore the mechanisms of autophagy-regulated huMSC function, Beclin 1 was knocked down, and the proteins associated with autophagy (Beclin 1, ATG7, LC3, P62), migration and differentiation (stromal cell-derived factor-1 (SDF-1)), stemness (Sox2), and apoptosis (caspase-3 and poly-ADP-ribose polymerase (PARP)) were examined with Western blot assay. The results showed that the expression of the autophagy pathway proteins Beclin 1, ATG7, LC3, and P62 were significantly decreased in the huMSCs-shBecn 1 group compared with those in the huMSCs-shNC group (Fig. 2a and b). This suggested that autophagy was precisely suppressed in huMSCs. SDF-1 plays a key role in the migration and differentiation of MSCs [34], and Sox2 as a transcriptional factor is essential for maintaining self-renewal/proliferation/pluripotency of undifferentiated stem cells [35]. Our data demonstrated that the inhibition of autophagy markedly reduced the expressions of SDF-1 and Sox2 in the huMSCs-shBecn 1 group (Fig. 2a and c). Furthermore, inhibition of autophagy can promote the expression of the apoptotic proteins caspase-3 and PARP in huMSCs (Fig. 2a and d).

To investigate the relationship between autophagy and CCaspase-3 and Sox2, LC3 and CCaspase-3, and P62 and Sox2 were co-localized by double immunofluorescence staining, respectively. We observed that impaired autophagy led to increased apoptosis (Fig. 2e) and reduced pluripotency in huMSCs (Fig. 2f).

These data indicated that autophagy is essential for maintaining huMSC function, including migration, differentiation, stemness, and survival.

### The effect of huMSC transplantation on spatial working memory, LTP, and DEP

**Fig. 3:**
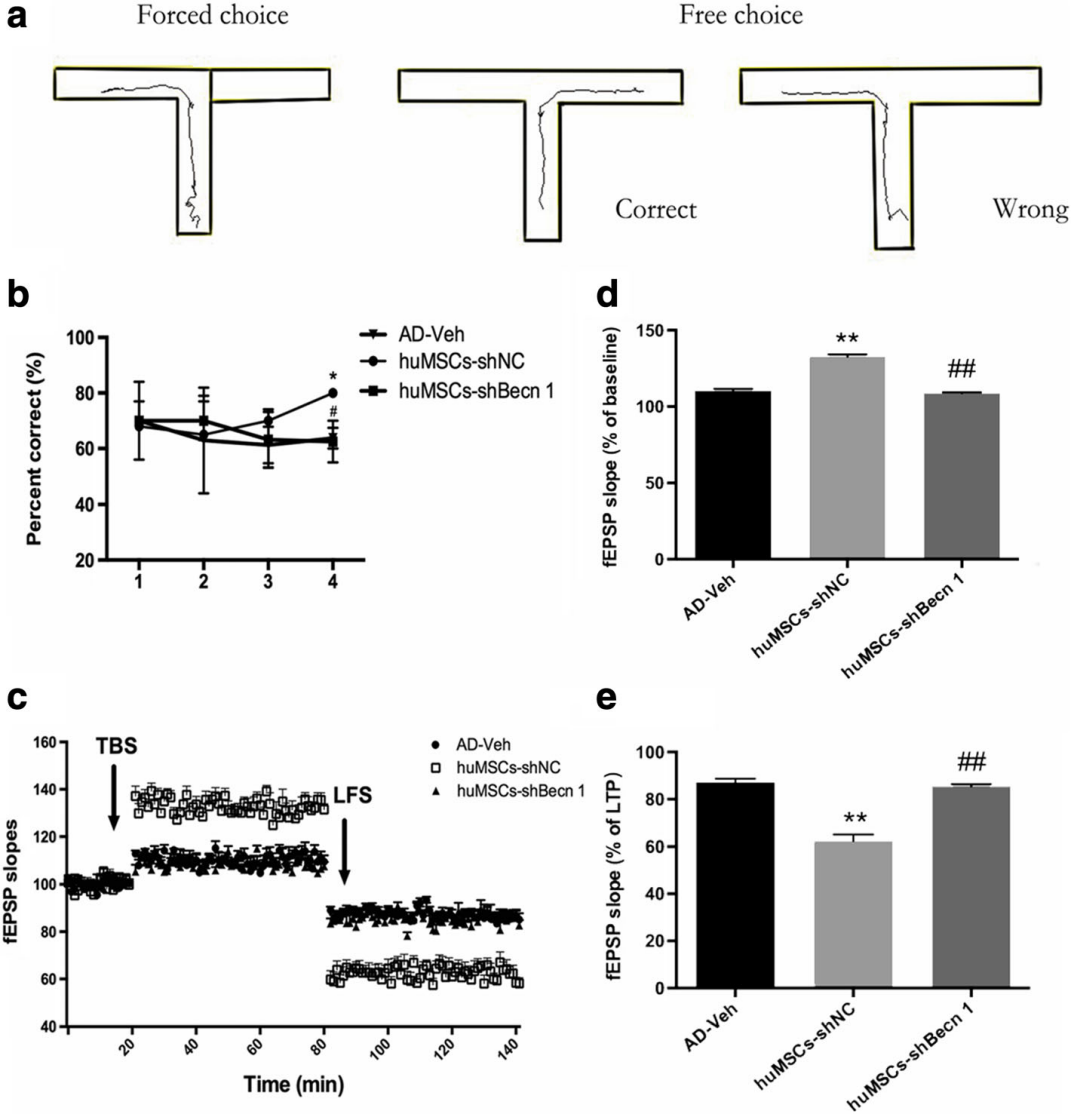
The effect of human umbilical cord mesenchymal stem cell (huMSC) transplantation on spatial working memory, LTP, and DEP. **a** T-maze task. **b** Mean percentage of correct responses for the rewarded alternation test on the elevated T-maze. **c** The response changes of long-term potentiation (LTP) and depotentiation (DEP) in the DG region. The first 20 min of evoked responses are the baseline. The theta burst stimulation (TBS) was used to induce LTP recording for 1 h, and low-frequency stimulation (LFS) was used to induce DEP recording for 1 h. **d** Mean field excitatory postsynaptic potential (fEPSP) slopes of LTP of all time points. **e** Mean fEPSP slopes of DEP of all time points. All data are expressed as mean ± SEM, *n* = 6 per group. *P < 0.05, **P < 0.01, vs. AD-Veh; ^#^*P* < 0.05, ^##^*P* < 0.01, vs. huMSCs-shNC

It is generally believed that AD is commonly characterized by a progressive learning and memory impairment. To assay the working memory performance of huMSC transplantation in an AD model mouse, a T-maze task was employed. In this task, the AD-Veh and huMSCs-shBecn 1 groups showed profound spatial working memory impairment. In the free choice phase, they failed or delayed the alternate response on two-choice mazes. Even at the end of the testing, they were still at accidental levels (percent correct: AD-Veh, 64%; huMSCs-shBecn 1, 63%). In contrast, in the huMSCs-shNC group the spatial response was improved from trial to trial and they obtained a gradual choice accuracy level of 80% (Fig. 3b).

LTP assays are a key indicator for estimating learning and memory. fEPSPs were evoked in the PP-DG region of the hippocampus pathway. After TBS stimulation, the fEPSP slopes increased abruptly from baseline in the huMSCs-shNC group, and the mean fEPSP slopes reached 132% of baseline. In contrast, the fEPSP slopes were not obviously increased with TBS stimulation in the AD-Veh and huMSCs-shBecn 1 groups (mean fEPSP slopes: AD-Veh, 110%; huMSCs-shBecn 1, 108%) (Fig. 3c and d). DEP assays are an index of reversal learning behavior, and are the opposite of LTP. DEP was stimulated with LFS and the mean slope of LTP was normalized and used as the baseline of DEP in each group. The data showed that DEP was significantly suppressed in the AD-Veh and huMSCs-shBecn 1 group (mean fEPSP slopes: AD-Veh, 87%; huMSCs-shBecn 1, 85%), but the DEP of the huMSCs-shNC group displayed good flexibility and the fEPSP slope dropped to 62% (Fig. 3c and e).

These results indicated that inhibition of autophagy in huMSCs to transplant could not ameliorate the impaired learning and memory in APP/PS1 transgenic mouse.

### The effect of huMSC transplantation on Aβ clearance in the cortex and hippocampus

**Fig. 4:**
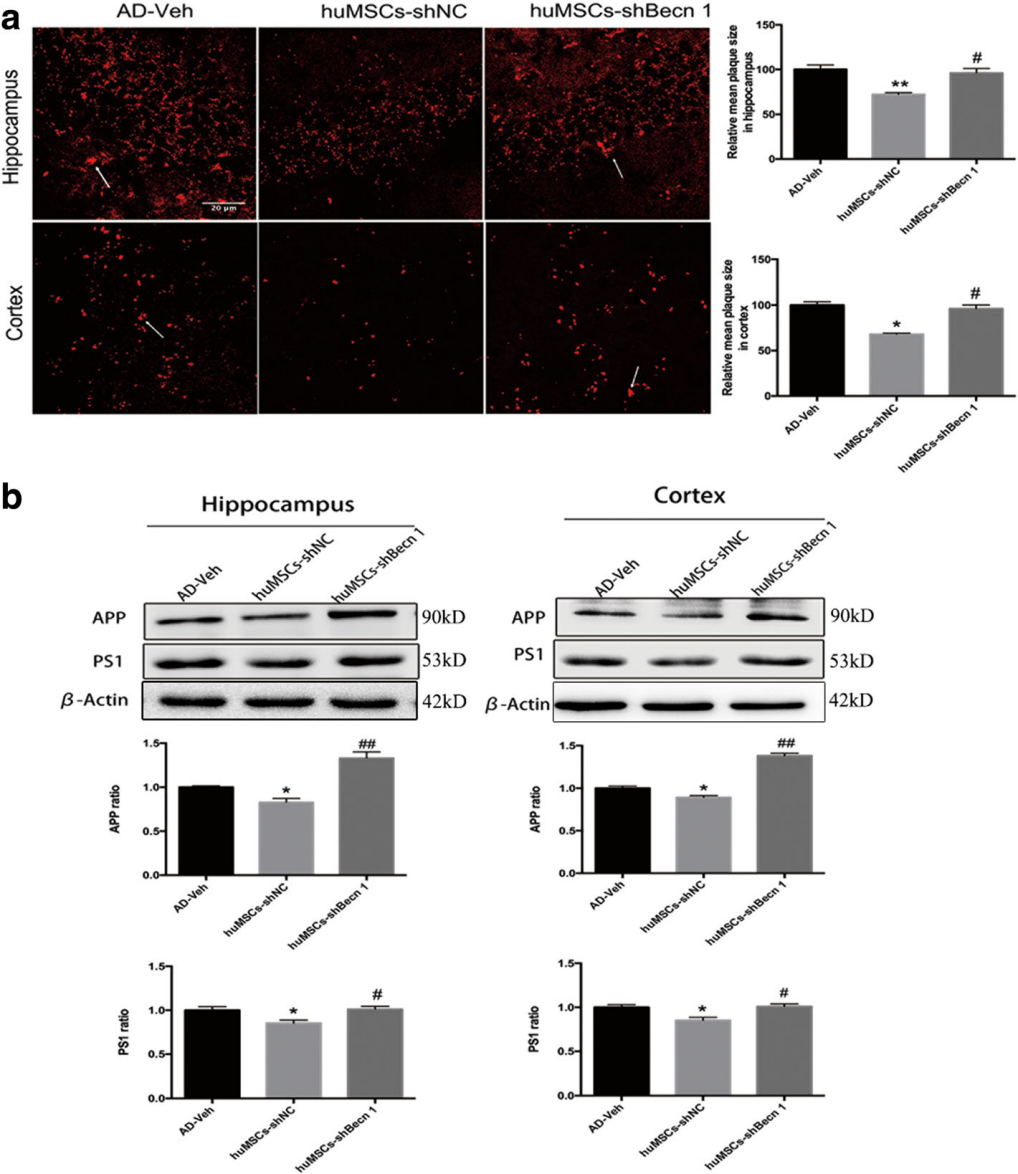
The effect of human umbilical cord mesenchymal stem cell (huMSC) transplantation on Aβ clearance in the cortex and hippocampus. **a** Aβ was detected by immunofluorescence staining and quantitative analysis in the cortex and hippocampus region (arrows indicate Aβ plaques). **b** Representative cropped Western blots and statistical analysis of amyloid precursor protein (APP) and presenilin 1 (PS1) in the cortex and hippocampus region. All data are expressed as mean ± SEM, *n* = 3 per group. Data were generated from three independent experiments. *P < 0.05, **P < 0.01, vs. AD-Veh; ^#^*P* < 0.05, ^##^*P* < 0.01, vs. huMSCs-shNC

To examine whether huMSC transplantation exhibited the ability to clear Aβ, we immunofluorescence stained Aβ with specific antibodies in the cortex and hippocampus region. The data analysis showed that the Aβ plaque significantly degraded in the huMSCs-shNC group compared with the AD-Veh and huMSCs-shBecn 1 groups (Fig. 4a). APP and PS1 protein levels, which lead to Aβ production, were then examined by Western blot assay. The levels of APP and PS1 were substantially reduced in the huMSCs-shNC group compared with the AD-Veh group. However, inhibition of autophagy in huMSCs failed to reduce the expression of APP and PS1 (Fig. 4b).

### The effect of huMSC transplantation on neuronal apoptosis of the cortex and hippocampus

**Fig. 5:**
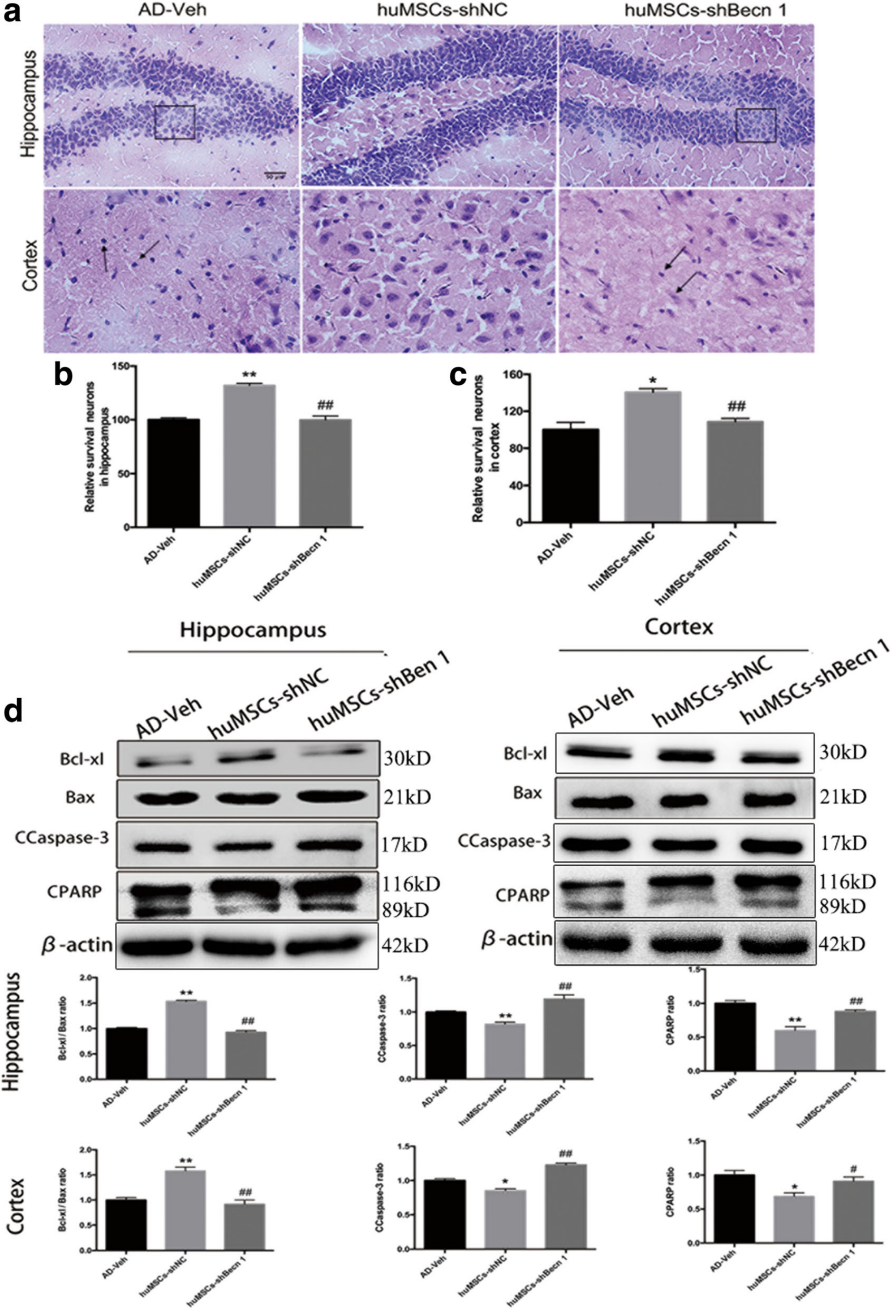
The effect of human umbilical cord mesenchymal stem cell (huMSC) transplantation on neuronal apoptosis of the cortex and hippocampus. **a** Cell apoptosis was evaluated by H&E staining. The boxes indicate neurons arranged loosely and neuron loss. The arrows indicate nucleus shrinkage. Relative number of surviving neurons in **b** the cortex and **c** the hippocampus. **d** Representative cropped Western blots and statistical analysis of the apoptosis-related proteins Bcl-xl, Bax, cleaved caspase-3 (CCaspase-3), and cleaved poly-ADP-ribose polymerase (CPARP) in the cortex and hippocampus. All data are expressed as mean ± SEM, *n* = 3. Data were generated from three independent experiments. *P < 0.05, **P < 0.01, vs. AD-Veh; ^#^*P* < 0.05, ^##^*P* < 0.01, vs. huMSCs-shNC

For the analysis of neuronal apoptosis in the cortex and DG of the hippocampus, H&E staining was employed. As shown in Fig. 5a, neurons in the AD-Veh and huMSCs-shBecn 1 groups were arranged loosely, with obvious nucleus shrinkage and neuron loss in the cortex and DG region. However, neurons in the huMSCs-shNC group were arranged in an orderly and dense manner. The number of surviving neurons was counted by ImageJ in each group, and the results showed that there were significantly more of these in the huMSCs-shNC group compared with the AD-Veh and huMSCs-shBecn 1 groups (Fig. 5b, c). To further understand the mechanism of apoptosis, proteins associated with apoptosis (Bcl-xl, Bax, CCaspase-3, and PARP) in the cortex and hippocampus lysates were examined by Western blot. The results showed that the ratio of Bcl-xl/Bax in the huMSCs-shNC group was higher than that in the AD-Veh and huMSCs-shBecn 1 groups, and that huMSCs-shNC significantly inhibited the expression of the apoptotic CCaspase-3 and PARP compared with the huMSCs-shBecn 1 group (Fig. 5d).

Thus, inhibition of autophagy in huMSCs blocked its anti-apoptosis function in the APP/PS1 transgenic mouse brain.

### Migration, apoptosis, and differentiation of huMSCs following transplantation in the APP/PS1 transgenic mouse

**Fig. 6:**
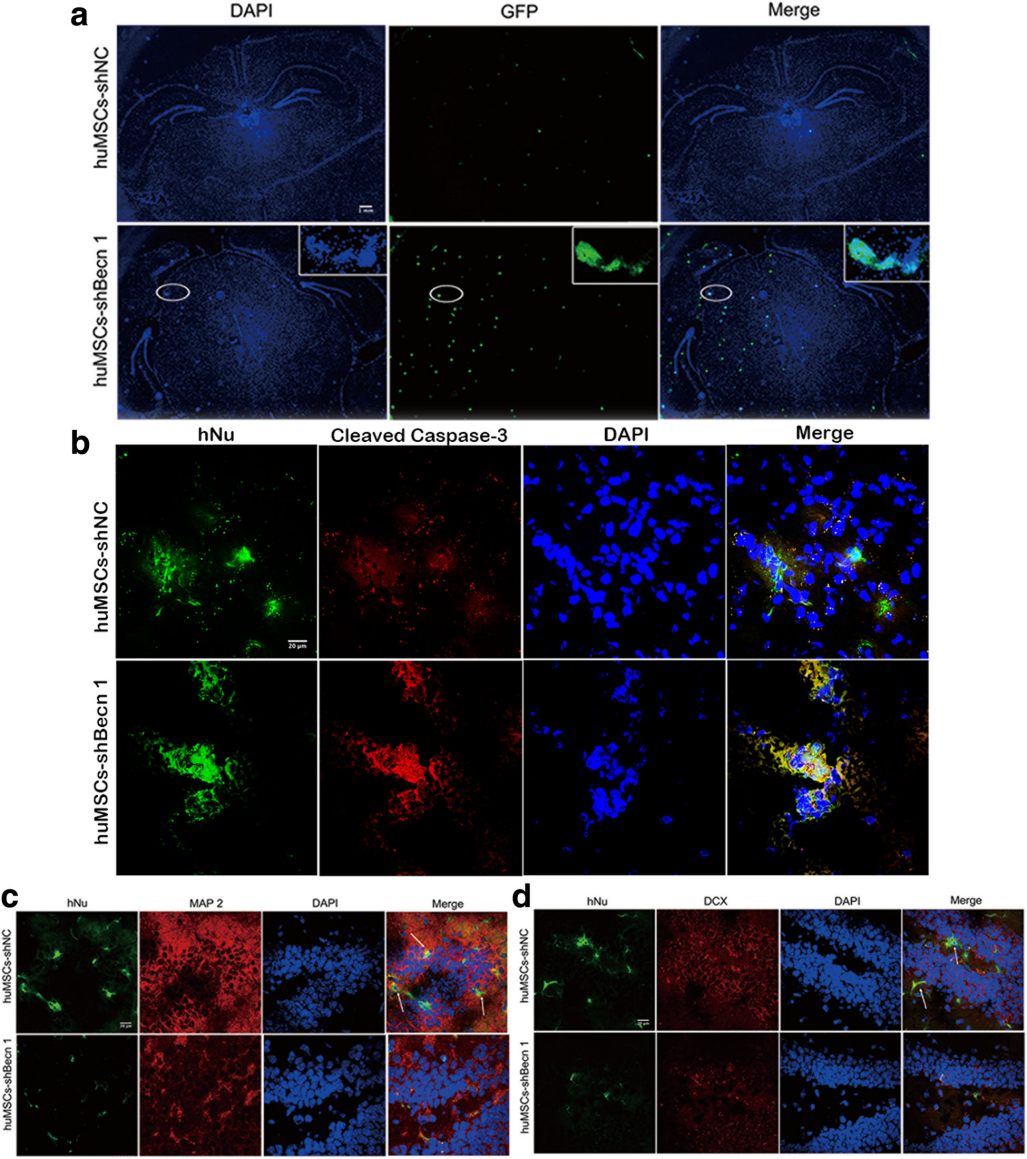
Migration of human umbilical cord mesenchymal stem cells (huMSCs) following transplantation in the APP/PS1 transgenic mouse. **a** Labeled green fluorescent protein (GFP) huMSC distribution in the whole brain. **b** Human nuclear (hNu) and cleaved caspase-3 were co-localized by double immunofluorescence staining in the cortex. **c** hNu and microtubule-associated protein 2 (MAP2) and **d** hNu and doublecortin (DCX) were co-localized by double immunofluorescence staining in the DG of the hippocampus

As shown in Fig. 6, labeled GFP huMSCs were observed at 14 days post-transplantation. huMSCs-shNC were widely distributed in the whole brain, but the majority of huMSCs-shBecn 1 were located in the left side of the brain and gathered into groups (Fig. 6a). The gathered blocks were labeled with CCaspase-3 antibody, and the expression of CCaspase-3 was obviously increased in the huMSCs-shBecn 1 group compared with the huMSCs-shNC group (Fig. 6b).

To investigate the differentiation of huMSCs in vivo, the newly born neuron marker doublecortin (DCX) and the mature neuron marker MAP2 were detected. The results indicated that huMSCs-shNC expressed DCX and MAP2 at high levels and, in turn, huMSCs-shBecn 1 did not (Fig. 6c and d).

These data showed that the ability of huMSCs, which migrated to the injury site and differentiated neurons, was blocked by inhibiting autophagy, consistent with the in vitro data.

### The effect of huMSC transplantation on neurogenesis of the subgranular zone (SGZ) and the subventricular zone (SVZ)

**Fig. 7:**
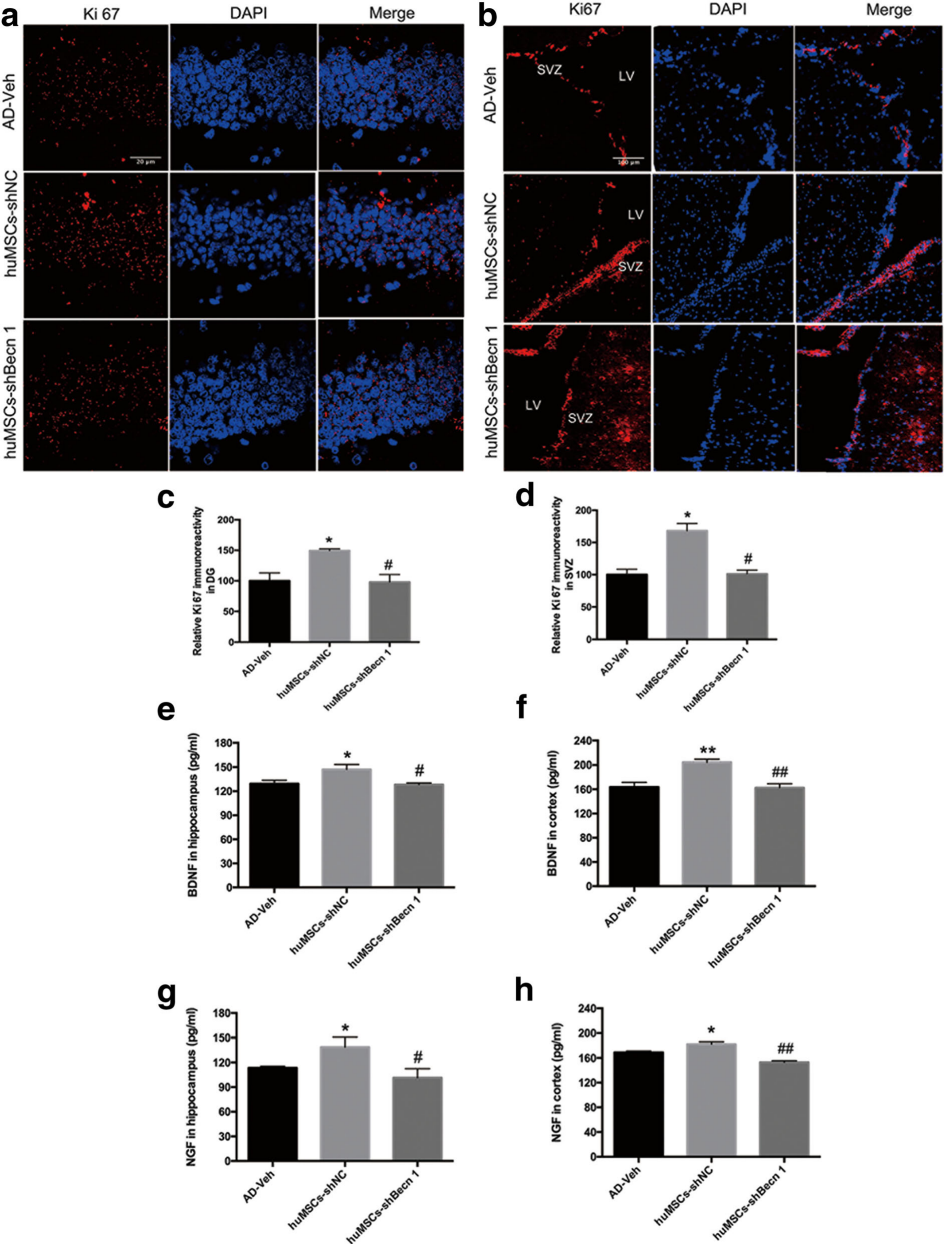
The effect of human umbilical cord mesenchymal stem cell (huMSC) transplantation on neurogenesis of the subgranular zone (SGZ) and the subventricular zone (SVZ). **a**–**d** Ki67 was detected by immunofluorescence staining and quantitative analysis by image J software in the SGZ and SVZ, respectively. **e**–**h** The effect of huMSC transplantation on brain-derived neurotrophic factor (BDNF) and nerve growth factor (NGF) secretion in the cortex and hippocampus by ELISA. All data are expressed as mean ± SEM, *n* = 3. Data were generated from three independent experiments. *P < 0.05, **P < 0.01, vs. AD-Veh; ^#^*P* < 0.05, ^##^*P* < 0.01, vs. huMSCs-shNC. DG dentate gyrus, LV lateral ventricle

In the adult brain, neurogenesis allows for continuous development under physiological and pathological stimuli. Neural stem cells (NSCs) are mostly located in the SGZ of the DG and the SVZ [36]. In this study, the proliferation marker Ki67 in the SGZ and SVZ was analyzed in APP/PS1 transgenic mice. The number of Ki67-positive cells in the huMSCs-shNC group was significantly increased compared with the AD-Veh group. However, in the huMSCs-shBecn 1 group, inhibition of huMSC autophagy failed to promote neurogenesis, and the number of Ki67-positive cells was equally matched with those of the AD-Veh group (Fig. 7a–d).

Furthermore, we measured the levels of the neurotrophic factors BDNF and NGF, which contribute to neurogenesis, by ELISA. The data suggested that the levels of BDNF and NGF significantly increased in the huMSCs-shNC group compared with the AD-Veh group, but the inhibition of autophagy in huMSCs could not promote the secretion of BDNF and NGF in the brains of APP/PS1 transgenic mice compared with that in the huMSCs-shNC group (Fig. 7e–h).

### The effect of huMSC transplantation on synaptic transmission

**Fig. 8:**
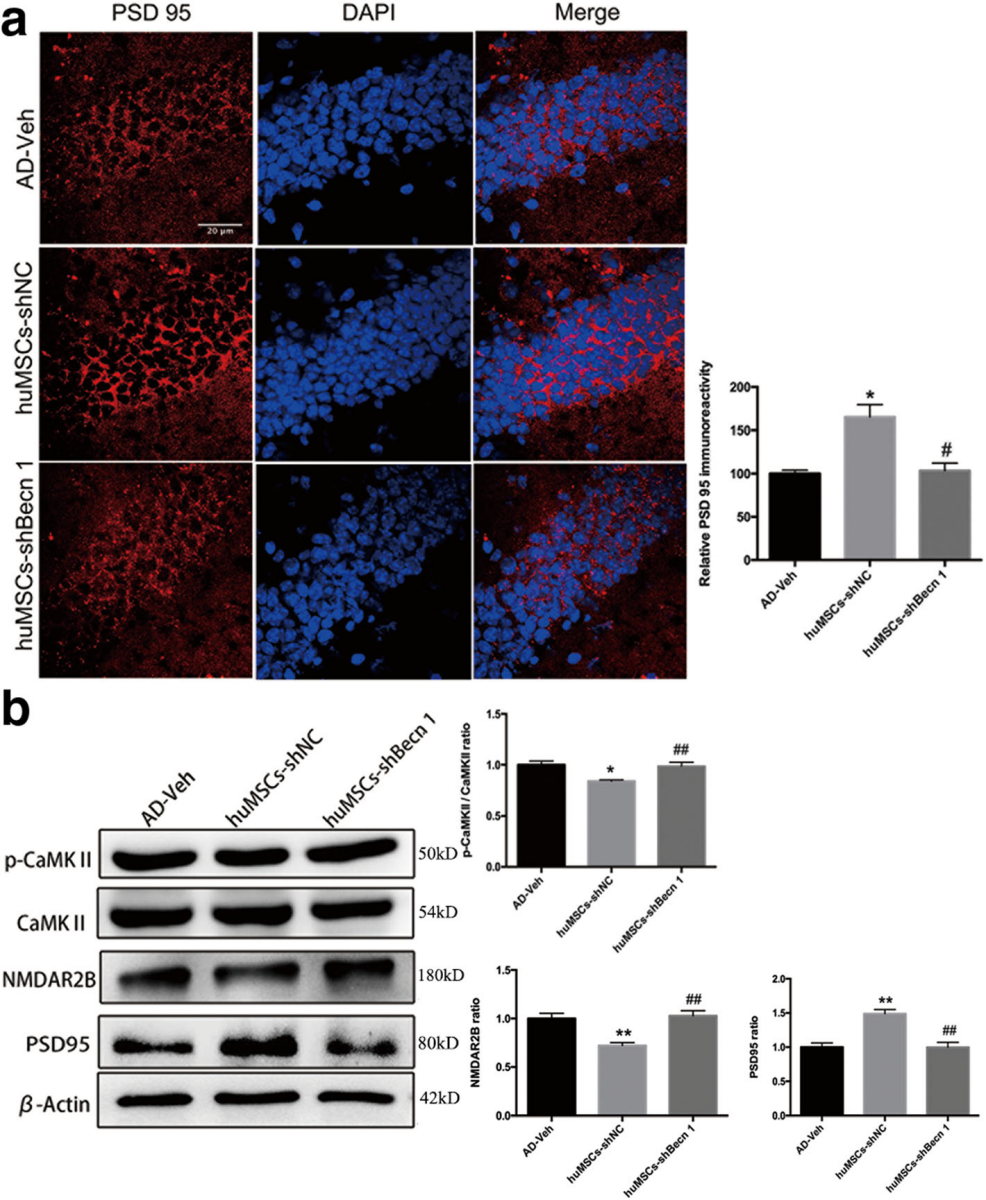
The effect of human umbilical cord mesenchymal stem cell (huMSC) transplantation on synaptic transmission. **a** PSD-95 was detected by immunofluorescence staining and quantitative analysis by ImageJ software in the DG of the hippocampus. **b** Representative cropped Western blots and statistical analysis of synaptic transmission-related proteins CaMKII, p-CaMKII, NMDAR 2B, and PSD95 in the hippocampus. All data are expressed as mean ± SEM, *n* = 3. Data were generated from three independent experiments. *P < 0.05, **P < 0.01, vs. AD-Veh; ^##^*P* < 0.01, vs. huMSCs-shNC

PSD-95 is an important factor that contributes to synaptic formation [37]. We next performed immunoreactivity studies to explain the functional recovery in the APP/PS1 transgenic mouse after huMSC transplantation, and focused on the possible links between neurogenesis and synapse formation. Immunoreactivity images showed a higher intensity of PSD-95 in the DG of the hippocampus in the huMSCs-shNC group compared with the AD-Veh and huMSCs-shBecn 1 groups (Fig. 8a). In addition, we checked the synaptic transmission-related proteins CaMKII, p-CaMKII, NMDAR 2B, and PSD95, which contributed to LTP generation in the hippocampus as seen by Western blot. The results indicated that the ratio values of p-CaMKII/CaMKII and NMDAR 2B were significantly decreased in the huMSCs-shNC group compared with the AD-Veh group. huMSCs-shBecn 1 transplantation did not produce a meaningful improvement. The level of PSD95 was significantly increased in the huMSCs-shNC group compared with the AD-Veh and huMSCs-shBecn 1 groups, consistent with the immunoreactivity data (Fig. 8b).

These data indicated that inhibition of autophagy in huMSCs failed to restore synaptic transmission injury in APP/PS1 transgenic mice.

## Discussion

Autophagy is crucial for regulating the stemness maintenance, expansion, and differentiation of stem cells [38]. Previous studies suggested that cultured MSCs continuously kept a high level of autophagy to maintain stemness [39, 40], and activating autophagy can promote MSC differentiation by various signaling pathways [41]. Moreover, activating autophagy can also block MSC apoptosis [42]. Based on the above, in this study we aimed to investigate the effect of autophagy in huMSCs. In vitro we found that migration of huMSCs was regulated by autophagy, and that activation of autophagy with Rap or inhibition with 3MA promoted or prevented the migration of huMSCs to the wound (Fig. 1a). We next examined the effect of autophagy on huMSC differentiation. huMSC-derived neurons were induced with D609 (Fig. 1b), and they showed a high level of the neuronal markers NSE and MAP2 (Fig. 1d). Moreover, autophagy was activated in the process of differentiation as indicated by the levels of LC3 at 2 h and 4 h (Fig. 1c). LC3 and MAP2 were then co-localized by immunofluorescence labeling (Fig. 1e) and further cell lysates, collected from Rap- or 3MA-treated huMSCs-NCs, were analyzed (Fig. 1f–h). These results were consistent and suggested that the level of autophagy was increased during huMSC differentiation, and that activation or inhibition of autophagy promoted or suppressed their differentiation.

To ascertain how autophagy controls huMSCs, Beclin 1 plays a central role in mediating the localization of other autophagy-related proteins to the phagophore membrane in autophagy [43]. Beclin 1 was downregulated in huMSCs by lentiviral transfection. We performed a huMSCs-shBecn 1 lysate analysis and found that, under conditions where autophagy was precisely suppressed as indicated by downregulation of Beclin 1, ATG7, LC3, and P62 expression (Fig. 2a and b), SDF-1 and Sox2 were reduced and represented the level of huMSC migration and stemness maintenance, respectively (Fig. 2c). In addition, downregulation of Beclin 1 led to increased apoptosis in huMSCs, which correlated with increased CCaspase-3 and CPARP (Fig. 2d). These results were consistent with those from immunostaining (Fig. 2e, f), and showed that autophagy influenced huMSC function via a variety of specific pathways.

Based on the above in vitro experiments, we further studied the effects of autophagy on huMSC transplantation in APP/PS1 transgenic mice used as an AD model. AD is characterized by massive neuronal death caused by Aβ plaques and tangle formation, and cognitive impairment caused by synaptic loss in several brain regions [21]. Previous research has suggested that stem cell transplantation could have a therapeutic potential for AD via various mechanisms. This is consistent with our results, as we found that huMSCs-shNC transplantation could improve the learning and memory ability of the mouse AD model by inhibition of neuronal apoptosis, promoting neurogenesis and synapse formation. However, and more importantly, inhibition of huMSC autophagy by knocking down Beclin 1 expression did not allow these neuroprotective effects in the mouse AD model.

The mouse AD model was associated with impairment of spatial working memory, which was managed by the hippocampus LTP [44]. Moreover, LTP is responsible for synaptic transmission. In this study, we found that huMSCs-shNC transplantation could ameliorate the spatial working memory (Fig. 3b), and the mean fEPSP of LTP was obviously enhanced in the mouse AD model (Fig. 3c, d). DEP is the opposite to LTP, responsible for balance between synaptic attenuation and enhancement, and involved in the forgetting and storage of information [45]. The mean fEPSP of DEP was reduced after huMSCs-shNC transplantation in the mouse AD model (Fig. 3c, e). In contrast, huMSCs-shBecn 1 transplantation could not restore the impaired working memory, and remained at the same level as the AD-Veh group. These findings suggested that autophagy is essential for huMSC neuroprotection.

We also demonstrated that huMSC-shNC transplantation could reduce Aβ production (Fig. 4a) and cell death (Fig. 5a–c) in the transgenic mouse brain. We identified the molecular mechanisms involving APP and PS1 (Fig. 4b) which related to Aβ peptide synthesis [46], and cell apoptosis-related proteins Bcl-xl, Bax, CCaspase-3, and PARP were decreased (Fig. 5d). However, inhibition of huMSC autophagy could not play a useful role in the mouse AD model.

To explore why transplanted huMSCs-shBecn 1 could not play a neuroprotective effect, we observed that most of the transplanted huMSCs-shBecn 1 were located in the left brain and clustered into groups. Transplanted huMSCs-shNC could extensively migrate, however, and aggregation rarely occurred (Fig. 6a). Furthermore, the gathered cells had undergone significant apoptosis as indicated by CCaspase-3 expression (Fig. 6b). Using the specific antibodies anti-hNu with DCX or MAP2 co-localization, we found that huMSCs-shNC migrated to the DG region of the hippocampus and differentiated into neurons. However, we found hardly any huMSCs-shBecn 1 in the DG region (Fig. 6c, d). We summarized that inhibition of autophagy in huMSCs caused them to fail to migrate to the damaged hippocampus, which was then related to cognitive dysfunction and further to neuronal differentiation. Furthermore, extensively distributed huMSCs-shNC promoted neurogenesis (Ki67) in SGZ and SVZ by increasing BDNF and NGF secretion in the transgenic mouse brain (Fig. 7), and due to inhibition of huMSC autophagy causing migration defects and increased apoptosis, huMSCs-shBecn 1 transplantation could not promote the neurogenesis.

‬In conjunction with the cognitive impairment and reduced LTP, a significant loss of synapses was found in the AD mouse model. In the AD mouse model, Aβ accumulation induced increased NMDAR2B expression [47, 48], and huMSCs-shNC transplantation blocked CaMKII phosphorylation and inhibited the expression of NMDAR2B. PSD-95 is a molecular partner with NMDAR, and forms a molecular complex to contribute to synaptic formation. We confirmed that huMSCs-shNC transplantation promoted the synapse formation by enhancing PSD-95 expression in the AD mouse model; when huMSC autophagy was inhibited, all of the above functions ceased (Fig. 8).

## Conclusions

**Fig. 9:**
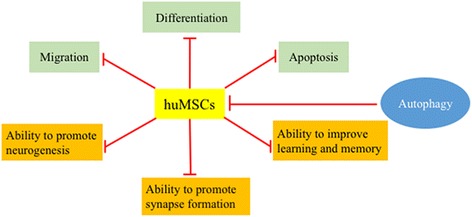
Inhibition of autophagy in huMSCs leads to its dysfunction, including migration, differentiation and anti-apoptosis, and promoting neurogenesis and synapse formation

Our results clearly show that autophagy dominates huMSC function and that inhibition of autophagy in huMSCs leads to the disappearance of functions including migration, differentiation, and anti-apoptosis, and the promotion of neurogenesis and synapse formation in the AD mouse model (Fig. 9). In conclusion, autophagy is required for huMSCs to maintain their function and improve cognition impairment in APP/PS1 transgenic mice. Our findings suggest that the therapeutic effect of huMSCs can be improved by increasing the level of autophagy, such as with traditional Chinese medicine or small molecule drug intervention. In addition, MSC senescence is also an important factor affecting its function, and autophagy is impaired in MSC senescence [49]. Therefore, a clear relationship between autophagy and senescence can also help researchers to improve the therapeutic effect of MSCs.

## Abbreviations

3MA: 3-Methyladenine
AD: Alzheimer’s disease
APP: Amyloid precursor protein
Aβ: Amyloid-β
BDNF: Brain-derived neurotrophic factor
D609: Tricyclodecane-9-yl-xanthogenate
DCX: Doublecortin
DEP: Depotentiation
DG: Dentate gyrus
DMEM: Dulbecco’s modified Eagle’s medium
ELISA: Enzyme-linked immunosorbent assay
FBS: Fetal bovine serum
fEPSP: Field excitatory postsynaptic potential
GFP: Green fluorescent protein
H&E: Hematoxylin and eosin
huMSC: Human umbilical cord mesenchymal stem cell
LFS: Low-frequency stimulation
LTP: Long-term potentiation
MAP2: Microtubule-associated protein 2
MSC: Mesenchymal stem cell
NC: Negative control
NGF: Nerve growth factor
NSE: Neuron-specific enolase
PARP: Poly-ADP-ribose polymerase
PBS: Phosphate-buffered saline
PP: Performant pathway
PS1: Presenilin 1
Rap: Rapamycin
SDF-1: Stem cell-derived factor-1
SGZ: Subgranular zone
shRNA: Small-hairpin RNA
SVZ: Subventricular zone
TBS: Theta burst stimulation
